# Therapeutic strategies in intracerebral hemorrhage: from hematoma stabilization to alleviating secondary brain injury

**DOI:** 10.1016/j.rpth.2026.103423

**Published:** 2026-03-26

**Authors:** Pei-Feng Hsieh, Yen-Bo Liu, Charlotte Cordonnier, Hsin-Hsi Tsai

**Affiliations:** 1 Department of Neurology, National Taiwan University Hospital, Taipei, Taiwan; 2 Department of Neurology, National Taiwan University Hospital Hsin-Chu Branch, Hsin-Chu, Taiwan; 3 Graduate Institute of Clinical Medicine, National Taiwan University College of Medicine, Taipei, Taiwan; 4 Division of Neurosurgery, Department of Surgery, National Taiwan University Hospital, Yunlin Branch, Yunlin County, Douliu, Taiwan; 5 University of Lille, Inserm, CHU Lille, U1172—LilNCog—Lille Neuroscience & Cognition, Lille, France

**Keywords:** hematoma, inflammation, intracerebral hemorrhage, neurosurgery, therapeutics

## Abstract

A state-of-the-art lecture titled, “New Treatment Targets in Intracerebral Hemorrhage,” was presented at the International Society on Thrombosis and Haemostasis (ISTH) Congress in 2025. Intracerebral hemorrhage remains one of the most devastating forms of stroke, with high rates of mortality and long-term disability. Unlike ischemic stroke, treatment options remain limited, but advances in pathophysiological understanding and clinical trials have expanded the range of potential therapeutic strategies. This review synthesizes current evidence across 3 major interdependent domains. First, hematoma stabilization focuses on preventing hematoma expansion through intensive blood pressure control, hemostatic agents, and reversal of antithrombotic therapies, with recent prehospital and care bundle trials demonstrating promise. Second, strategies to reduce hematoma burden and the mass effect include both surgical evacuation and enhancement of endogenous clearance mechanisms. While minimally invasive surgery has shown selective benefits in carefully chosen patients, increasing attention is being directed toward accelerating natural hematoma resolution via microglial and macrophage-mediated phagocytosis, as well as modulating iron toxicity. Third, mitigation of secondary brain injury targets perihematomal edema, oxidative stress, and neuroinflammation. Recent trials of glibenclamide, anti-inflammatory agents, COX-2 inhibitors, and antioxidants have indicated both the challenges and opportunities of modulating secondary injury. Together, these developments represent a transition from therapeutic nihilism toward optimism but emphasize a need for multimodal and time-sensitive interventions. Finally, we summarize relevant new data on this topic presented during the 2025 ISTH Congress.

## Essentials

• Intracerebral hemorrhage (ICH) is a severe stroke with few treatments and high mortality.
• This review highlights new ways to stabilize bleeds, remove clots, and protect the brain.
• Timely, multimodal care bundles are replacing the old view that nothing can be done for ICH.
• Minimally invasive surgery and tailored treatments are now improving patient outlooks.

## 1 Introduction

Intracerebral hemorrhage (ICH) remains one of the most devastating forms of stroke, with a 30-day mortality rate of ∼40% and substantial long-term disability among survivors [1]. In contrast to ischemic stroke, treatment options for ICH are limited, and most interventions have failed to demonstrate clear functional benefits in large clinical trials. Nonetheless, emerging evidence suggests that timely, targeted interventions can improve the outcomes of patients with ICH.

ICH results in both primary and secondary brain injuries. Primary mechanical injury occurs immediately due to direct tissue destruction and disruption of white matter tracts. In the acute phase, hematoma expansion (HE) can exacerbate this injury. The mass effect of the hematoma may also increase intracranial pressure and impair cerebral perfusion. Within days, secondary injury is driven by neurotoxic products of blood degradation, cerebral edema, neuroinflammation, and oxidative stress, all of which contribute to subsequent neurologic damage [2].

**Figure 1:**
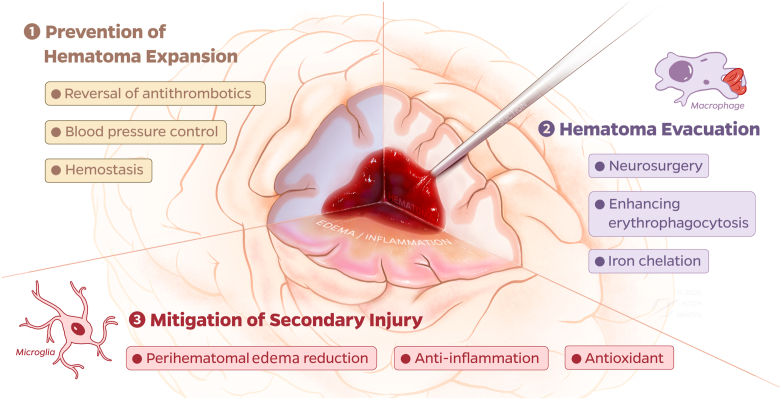
Schematic illustration of the 3 biologically interlinked stages in intracerebral hemorrhage management. Stage 1: prevention of hematoma expansion through early blood pressure control, hemostatic therapy, and reversal of antithrombotic agents. Stage 2: reduction of hematoma burden via surgical evacuation and enhancement of endogenous clearance pathways and iron chelation, which represent emerging therapeutic focuses. Stage 3: mitigation of secondary brain injury by targeting perihematomal edema, oxidative stress, and neuroinflammation, with ongoing investigations into edema-reducing agents, anti-inflammatory strategies, and antioxidant therapies.

From a therapeutic perspective, the management of ICH can be conceptualized into 3 stages, as illustrated in Figure 1: prevention of HE (stage 1), hematoma evacuation and reduction of the mass effect (stage 2), and mitigation of secondary brain injury, including perihematomal edema (PHE) and inflammation (stage 3). The major clinical trials that have investigated therapeutic strategies for these stages of ICH are summarized in Tables 1 (medical interventions) and 2 (surgical interventions). While these 3 stages are often discussed as distinct clinical phases, they represent a biologically interdependent continuum; consequently, the development of synchronized, multimodal treatment strategies is essential to improve the management of ICH.

**Table 1:** Medical (pharmacologic) interventions for ICH—a summary of key trials.

Agent/strategy	Trial (phase)	Patient population and case number	ID/status	Intervention vs comparator	Primary outcome	Result and adverse effects
TXA [3]	TICH-2 (III)	ICH <8 h; *n* = 2325	ISRCTN93732214/completed	TXA 1 g i.v. + 1 g infusion vs placebo	mRS at 90 d	No mRS improvement; ↓ HE; safe, no ↑ thromboembolism
TXA [4]	STOP-AUST (II)	Spot sign ICH <4.5 h; *n* = 100	NCT01702636/completed	TXA 1 g i.v. + 1 g infusion vs placebo	HE	No difference in expansion; no ↑ thromboembolism
TXA [5]	STOP-MSU (II)	Hyperacute ICH <2 h; *n* = 201	NCT03385928/completed	TXA 1 g i.v. + 1 g infusion vs placebo	HE	No difference in expansion; no ↑ thromboembolism
TXA	TICH-3 (III)	ICH <4.5 h; *n* = 5500 (ongoing)	ISRCTN97695350/recruiting	TXA 1 g i.v. + 1 g infusion vs placebo	Death at 7 d	Ongoing
rFVIIa [6]	FAST (II/III)	Spontaneous ICH <4 h; *n* = 821	NCT00127283/completed	rFVIIa (20 or 80 μg/kg) vs placebo	mRS ≥5 at 90 d	No functional benefit; ↓ hematoma growth; ↑ thromboembolic risk at high dose
rFVIIa [7]	SPOTLIGHT (II), STOP-IT (II)	Spot sign ICH; ICH <6.5 h; *n* = 69	NCT01359202/completed NCT00810888/completed	rFVIIa (80 μg/kg) vs placebo	HE	No significant difference in growth, mRS, or mortality
rFVIIa	FASTEST (III)	Ultraearly ICH <2 h; *n* = 860 (ongoing)	NCT03496883/recruiting	rFVIIa (80 μg/kg) vs placebo	Ordinal mRS	Ongoing^a^
Platelet transfusion [8]	PATCH (III)	ICH on antiplatelets; *n* = 190	NTR1303/completed	Platelet transfusion vs standard care	mRS 4-6 at 90 d	Worse outcomes with transfusion; ↑ serious adverse events
4-factor PCC [9]	INCH (III)	Warfarin-associated ICH <12 h and INR ≥2.0; *n* = 50	NCT00928915/completed	4-factor PCC vs FFP	INR ≤1.2 in 3 h and HE	Faster reversal with PCC; stopped early and underpowered for a clinical difference
Andexanet alfa [10]	ANNEXA-I (III)	DOAC Xa inhibitor-related ICH <15 h; *n* = 530	NCT03661528/completed	Andexanet alfa vs usual care	Hemostatic efficacy	Better control of HE; but higher thrombotic events
Pioglitazone [11]	SHRINC (II)	ICH <24 h; *n* = 84	NCT00827892/completed	Pioglitazone vs placebo	Edema volume and safety	No difference in edema/function; safe
RIC [12]	RESIST (III) ––ICH subgroup	Stroke including ICH; ICH *n* = 165	NCT03481777/completed	RIC vs sham	mRS shift and mortality	No significant functional benefit or 90-d mortality reduction in ICH subgroup
Deferoxamine [13]	i-DEF (II)	ICH <24 h; *n* = 291	NCT02175225/completed	Deferoxamine 32 mg/kg/d × 3 d vs placebo	mRS ≤2 at 90 d	No 90-d benefit; post hoc 180-d signal; well-tolerated
Mannitol [14]	MACE-ICH (IIb)	ICH <72 h; *n* = 45 planned	ISRCTN15383301/recruiting	Mannitol vs standard care	Change in ICH and edema volume	Ongoing
Glibenclamide [15]	GATE-ICH (II)	ICH <72 h; *n* = 220	NCT03741530/completed	Glibenclamide vs placebo	Edema volume and mRS at 90 d	↓ Edema; trend toward improved outcomes; ↑ hypoglycemia
Minocycline [16]	MACH (I/II)	ICH <12 h; *n* = 16	NCT01805895/completed	Minocycline vs placebo	Anti-inflammatory profile	No difference in inflammatory biomarkers, hematoma volume, or PHE at 90 d
Minocycline [17]	MITCH (I/II)	ICH <12 h; *n* = 20	NCT03040128/completed	Minocycline vs placebo	Treatment adverse effects	No difference in clinical and radiological outcomes. Drug was associated with a decrease in MMP-9 level
Minocycline [18]	BATMAN (I/II)	CAA-ICH; *n* = 60 planned	NCT05680389/recruiting	Minocycline vs placebo	CSF inflammation profile	Ongoing
Anakinra [19]	BLOC-ICH (II)	ICH <8 h; *n* = 25	NCT03737344/completed	Anakinra vs placebo	Edema and mRS	Underpowered to assess clinical efficacy; mild injection reactions
Fingolimod	FITCH (I)	ICH <24 h; *n* = 28	NCT04088630/completed	Fingolimod 0.5 mg vs placebo	Edema volume and safety	Results pending; transient bradycardia noted
Celecoxib [20]	ACE-ICH (II)	ICH <24 h; *n* = 44	NCT00526214/completed	celecoxib 400 mg twice daily × 14 d vs usual care	PHE volume	↓ PHE expansion; no statistically mRS benefit
Celecoxib	-(II)	ICH <6 h; *n* = 60	NCT05434065/recruiting	Celecoxib 200 mg daily × 21 d vs usual care	HE and PHE volume	Ongoing
Colchicine	CoVasc-ICH (II)	ICH <48 h; *n* = 100	NCT05159219/completed	Colchicine vs placebo	Feasibility	Results awaited
Statin continuation	SATURN (III)	Prior statin, lobar ICH; *n* = 1456	NCT03936361/recruiting	Continue vs stop statin	ICH recurrence and CV events	Ongoing
Atorvastatin	STATIC (II/III)	Acute ICH <24 h; *n* = 98	NCT04857632/recruiting	Atorvastatin 20 mg/d × 7 d vs usual care	PHE and mRS	Ongoing

CAA, cerebral amyloid angiopathy; FFP, fresh frozen plasma; HE, hematoma expansion; ICH, intracerebral hemorrhage; INR, international normalized ratio; mRS, modified Rankin scale; PCC, prothrombin complex concentrate; PHE, perihematomal edema; rFVIIa, recombinant activated factor VII; RIC, remote ischemic conditioning; TXA, tranexamic acid.

a FASTEST trial was recently published. The findings suggested recombinant factor VIIa administered within 2 h of ICH onset slowed haematoma growth, but did not improve functional outcomes and showed a small increased risk of life-threatening thromboembolic complications.[PMID: 41653933].

**Table 2:** Surgical interventions for ICH—techniques, indications, and evidence.

Surgical method	Trial	Patient population and case number	NCT ID/status	Primary outcome(s)	Key findings
Open craniotomy [21]	STICH	40% lobar ICH, 40% deep ICH, and 20% both, *n* = 1033	ISRCTN19976990/completed	GOSE at 6 mo	No overall benefit; possible benefit to subgroup with lobar ICH ≤1 cm from the surface
Open craniotomy [22]	STICH II	Lobar ICH, superficial; *n* = 601	ISRCTN22153967/completed	GOSE at 6 mo	No overall benefit
MIS + thrombolysis [23]	MISTIE II	ICH ≥20 mL, stable ICH volume ≥6 h; *n* = 96	NCT00224770/completed	Safety outcomes: 30-d mortality	MIS + alteplase was safe
MIS + thrombolysis [24]	MISTIE III	ICH ≥30 mL, stable ICH volume ≥6 h; *n* = 499	NCT01827046/completed	mRS 0-3 at 1 y	↓ Mortality; no significant functional benefit, except in those with postsurgery volume of ≤15 mL
Endoscopic MIS [25]	ENRICH	Lobar or anterior basal ganglia ICH 30-80 mL; *n* = 300	NCT02880878/completed	mRS shift at 6 mo	Improved outcomes in surgical arm in lobar ICH
Decompressive craniectomy [26]	SWITCH	Large deep ICH >30 mL; *n* = 197	NCT02258919/completed	mRS 0-4 at 6 mo	Trend toward benefit; not statistically significant
IVH + EVD ± thrombolysis [27]	CLEAR III	IVH + hydrocephalus; *n* = 500	NCT00784134/completed	mRS 0-3 at 180 d	Irrigation with alteplase ↓ mortality, but ↑ severe disability

EVD, external ventricular drain; GOSE, Glasgow outcome scale-extended; ICH, intracerebral hemorrhage; IVH, intraventricular hemorrhage; MIS, minimally invasive surgery; mRS, modified Rankin scale.

## 2 Stage 1: Prevention of HE

**Figure 2:**
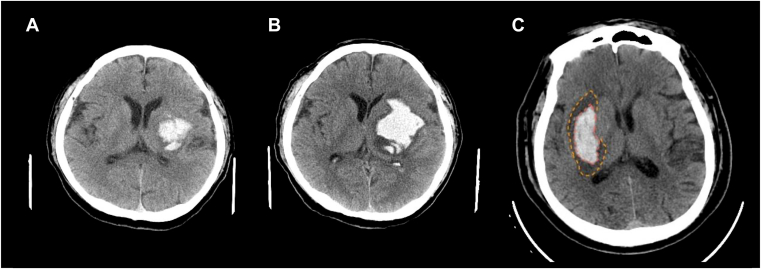
Hematoma expansion and perihematomal edema following intracerebral hemorrhage (ICH). (A) Day 1: left putaminal ICH with an initial volume of 18 mL. (B) Day 2: hematoma expansion to 50 mL. (C) In the days after ICH, perihematomal edema develops around the hematoma (outlined by red dashed lines for the hematoma and orange dashed lines for the edema).

HE occurs in ∼20% to 30% of patients (Figure 2A, B), mainly within the first 3 to 6 hours after onset, and is strongly associated with poor outcomes [28]. The operational definitions for HE include absolute growth of the hematoma to >6 mL, a relative increase in volume of >33%, or development of delayed intraventricular hemorrhage (IVH). The primary therapeutic goal in this phase is rapid stabilization of bleeding.

### 2.1 Blood pressure control

Early blood pressure control is a current cornerstone of medical intervention for many conditions, including ICH. Acute blood pressure elevation is common in ICH and is strongly linked to HE and PHE. The INTERACT2 trial compared intensive systolic blood pressure (SBP) lowering (<140 mm Hg within 1 hour) vs standard care (<180 mm Hg) among 2839 patients with ICH with SBP of 150 to 220 mm Hg, 84% of whom had deep ICH. The median baseline volume was 11 mL. Intensive treatment was safe and showed a trend toward improved functional outcomes at 90 days (adjusted odds ratio, 0.87, 95% CI, 0.75-1.01) [29]. The ATACH-II trial (*n* = 1000) investigated the effects of reducing SBP to 110 to 139 mm Hg vs 140 to 179 mm Hg within 4.5 hours using intravenous nicardipine; however, this approach offered no functional benefit and increased renal complications [30]. However, the INTERACT3 trial demonstrated that implementing a care bundle—including early intensive SBP lowering, glycemic control, pyrexia management, and anticoagulation reversal—was associated with improved functional outcomes [31].

More recently, the INTERACT4 trial (*n* = 2404) evaluated prehospital SBP lowering with intravenous urapidil within 2 hours in patients with confirmed ICH and demonstrated improved functional outcomes (common odds ratio, 0.75; 95% CI, 0.60-0.92) [32]. High SBP variability during the acute phase has also been associated with poor outcomes [33]. A meta-analysis of individual patient data from the INTERACT 1-4 trials indicated that early intensive blood pressure reduction was safe and associated with improved functional recovery, despite a nonsignificant effect in terms of reducing hematoma growth [34]. Based on this evidence, current recommendations advocate SBP should be lowered rapidly and safely to ∼140 mm Hg, ideally within the first 2 hours, while minimizing variability [35,36]. The recently published ICH ADAPT-2 trial showed that intensive SBP lowering (<140 mm Hg) did not increase the prevalence or volume of ischemic lesions on diffusion-weighted imaging compared with lowering to standard targets (<180 mm Hg) [37]. However, the optimal choice of antihypertensive agent and the duration of intensive blood pressure lowering remain uncertain; ideally, the drugs should be fast-acting and short-acting to achieve rapid, sustained control without excessive lowering [36].

### 2.2 Hemostatic and antifibrinolytic therapy

The use of hemostatic agents may be a reasonable therapeutic approach to limit HE, even in patients without acquired or congenital coagulation disorders. Tranexamic acid (TXA) has been evaluated in several trials. The TICH-2 trial (*n* = 2325) found that administration of TXA within 8 hours reduced HE but did not improve mortality or functional outcomes and did not increase thrombotic risk [3]. The STOP-AUST and STOP-MSU trials examined the use of TXA in patients with the spot sign (a risk factor that predicts HE) or ultraearly administration within 2 hours. Neither trial demonstrated a significant benefit [4,5]. The ongoing TICH-3 trial is designed to evaluate the use of TXA within 4.5 hours (ISRCTN97695350). Current guidelines reflect uncertainty regarding the clinical benefit of TXA, although expert opinion suggests TXA may be considered to reduce HE [36].

Recombinant activated factor VIIa (rFVIIa) reduced hematoma growth in the Factor Seven for Acute Hemorrhagic Stroke trial but did not improve functional outcomes and increased thromboembolic risk [6]. In the SPOTLIGHT and STOP-IT trials, administration of FVIIa to selected patients with a positive computed tomography spot sign within 6.5 hours showed no benefit in terms of reducing HE or improving clinical outcomes [7]. The ongoing recombinant factor VIIa for hemorrhagic acute stroke treatment at earliest possible time trial (NCT03496883) is assessing whether ultraearly administration of FVIIa, within 2 hours of onset, can improve clinical outcomes. At present, hemostatic therapy in acute ICH remains investigational [38].

### 2.3 Management of antithrombotic-associated ICH

ICH associated with use of antithrombotic agents (including antiplatelet and anticoagulants) represents up to 40% of cases of nontraumatic ICH [39]. For patients with antiplatelet-associated ICH, platelet transfusion did not reduce the risk of HE and may worsen functional outcomes; the use of this strategy should be avoided outside surgical contexts, where evidence is uncertain [8,40]. The phase II DASH feasibility trial investigated desmopressin in ICH associated with antiplatelet therapy and found no significant effect on mortality, dependency, HE, or thromboembolic events; thus, the clinical benefit of desmopressin remains unresolved [41].

Up to 20% of cases of ICH are related to anticoagulants and necessitate rapid reversal. In more than half of cases of vitamin K antagonist-associated ICH, the international normalized ratio (INR) is within the therapeutic range at admission [42]. The treatment goal is to achieve and maintain an INR <1.3 as rapidly as possible [35,36]. Prothrombin complex concentrate (PCC) achieves faster INR correction than fresh frozen plasma. The INCH trial was terminated early by the data safety monitoring board due to the clear superiority of PCC for INR normalization and was therefore underpowered to assess clinical outcomes [9]. In addition, immediate intravenous administration of vitamin K is recommended and may require repeat administration to prevent a rebound of the INR [36].

PCC is recommended for direct oral anticoagulant-related ICH, particularly patients on factor Xa inhibitors. However, randomized trial evidence is lacking, and observational studies have not demonstrated clear benefits in terms of reduced mortality, improved functional status, or lower rates of HE [43]. Specific reversal agents are also available. Andexanet alfa was evaluated in the ANNEXA-I trial and demonstrated superior hemostatic efficacy than PCC but was associated with an increased risk of thromboembolic events, without improvements in mortality or functional outcomes [10]. Similarly, the TICH-NOAC trial tested TXA in addition to PCC for direct oral anticoagulant–related ICH and found no benefit in terms of reducing hematoma size, functional outcomes, or mortality, although thromboembolic risk was not increased [44]. Idarucizumab, a monoclonal antibody fragment, achieves rapid and complete reversal of the FIIa inhibitor dabigatran, as shown in the REVERSE-AD study, although no randomized trial has specifically assessed the clinical outcomes of idarucizumab in ICH [45].

## 3 Stage 2: Hematoma Evacuation and Management of the Mass Effect

The primary rationale for surgical intervention in ICH is to alleviate the mass effect, reduce intracranial pressure, and mitigate secondary brain injury caused by the hematoma and its breakdown products. The choice of surgical technique depends on the location and size of the hematoma and associated complications. Current strategies include conventional craniotomy for hematoma evacuation, minimally invasive surgery (MIS) for hematoma evacuation, catheter-based thrombolysis, decompressive craniectomy, and procedures for IVH. The 2022 American Heart Association guidelines recommended surgical management of ICH to save lives in deteriorating patients but did not recommend systematic surgery to improve functional outcomes [35]. In 2025, the European Stroke Organization and European Association of Neurosurgical Societies conducted a meta-analysis of 17 randomized controlled trials (RCTs) of surgical approaches that aimed to achieve hematoma removal. The results of this meta-analysis showed that surgical intervention reduced the risk of death and may also improve functional outcomes [36].

### 3.1 Conventional craniotomy for hematoma evacuation

Craniotomy aims to directly remove the clot and relieve the mass effect. Large randomized trials, including STICH I and II, did not demonstrate an overall functional benefit of early surgical evacuation compared with initial medical management, although subgroup analyses suggested a possible mortality benefit in selected patients (eg, lobar ICH near the cortical surface) [21,22,46]. It is thought that the potential benefits of clot removal may have been negated by surgical tract injury. Recent propensity analyses of conventional craniotomy and various meta-analysis of RCTs have reported conflicting results, which suggests conventional craniotomy does not benefit patients with ICH [47,48]. To overcome the limitations of open surgery, minimally invasive surgical techniques for ICH evacuation have been developed.

### 3.2 MIS for hematoma evacuation

MIS approaches have attracted renewed interest, as accumulating evidence suggests that MIS may improve functional outcomes and reduce mortality [36,46,49]. Overall, the beneficial outcomes associated with MIS might be due to lower posttreatment volumes and earlier intervention [28]. MIS includes various approaches and is best understood as both a mindset and a technique that aim to reduce secondary iatrogenic injury and preserve tissue integrity while effectively reducing the hematoma volume. MIS integrates preoperative and intraoperative navigation, minimization of cortical incision, respect for eloquent tracts, and trajectory planning similar to the principles of tumor surgery [36].

Meta-analyses of older RCTs suggested stereotactic aspiration and endoscopic removal improved mortality, but most of these trials were underpowered and methodologically heterogeneous, and there remains a need for high-quality evidence [49]. In the recent MISICH RCT, both endoscopic surgery and stereotactic aspiration improved 6-month functional outcomes in hypertensive ICH, especially in cases with deep hemorrhages, compared with small bone flap craniotomy [50]. In a retrospective analysis of a real-world registry that used propensity score matching, MIS—including stereotactic surgical evacuation and endoscopic surgery—was associated with lower in-hospital mortality and more favorable status at discharge but not with improved ambulatory status or functional outcomes compared with no surgery [51].

Currently, several trials of MIS based on different novel neuroendoscopy/systems are ongoing [52]. In addition, some approaches have added thrombolysis (typically alteplase), which is administered through a stereotactically placed catheter to gradually reduce the clot burden. In the MISTIE II and III trials, this approach resulted in safe clot reduction but provided no overall functional benefit [23,24]. Importantly, exploratory analysis from MISTIE III found that achieving a residual hematoma volume of <15 mL was associated with a 10.5% absolute increase in a favorable outcome (modified Rankin scale [mRS], 0-3 at 1 year), which establishes <15 mL as a potential target for future surgical interventions [24]. However, meta-analysis of the MISTIE II, MISTIE III, and Stereotactic treatment of Intracerebral Hematoma by means of a Plasminogen Activator trials found MIS had no significant overall effect on mortality or function, suggesting the benefits may depend on aggressive volume reduction and procedural expertise [23,24,36,53].

Recently, the ENRICH (early minimally invasive removal of intracerebral hemorrhage) trial evaluated MIS with trans-sulcal parafascicular techniques within 24 hours for hematoma volumes of 30 to 80 mL. MIS improved the mean utility weighted-mRS score at 180 days in lobar ICH, but not in deep ICH. However, only 2.6% of screened patients were eligible, which limits the generalizability of this study [25].

### 3.3 Decompressive craniectomy

The recent SWITCH RCT assessed decompressive craniectomy within 72 hours of onset for severe deep ICH (basal ganglia or thalamus, volume 30-100 mL) vs best medical management in patients aged 18 to 75 years. The primary outcome—death or severe dependency (mRS, 5-6) at 6 months—favored decompressive craniectomy (relative risk, 0.77; 95% CI, 0.59-1.01; *P* = .057), but the difference was not statistically significant. Notably, surgery improved the rate of survival with moderate disability (mRS, 4) but did not increase the rate of favorable outcome (mRS, 0-3) [26]. For patients with very large deep hematomas and a life-threatening mass effect, decompressive craniectomy without clot evacuation remains a consideration; however, decisions should be guided by patient and family values, weighing survival against expected quality of life.

### 3.4 IVH extension

IVH extension with acute hydrocephalus is conventionally managed with external ventricular drain (EVD); adjunctive intraventricular thrombolysis has been evaluated in several trials, including CLEAR III [27,36]. A meta-analysis showed that intraventricular thrombolysis reduced mortality but increased the rate of survival with severe disability (mRS, 5), with no improvement in functional independence [27,36]. These unsatisfying results make the implementation of thrombolysis protocols difficult in clinical practice. In addition, no adequately powered RCT has yet compared endoscopic clot evacuation with EVD vs EVD alone in patients with IVH. The meta-analysis of current available trials suggested thrombolysis may possibly improve functional outcomes and lead to lower shunt dependency; however, there is substantial heterogeneity between studies and a lack of adequately powered trials [36].

### 3.5 Endogenous hematoma clearance

Significant spontaneous hematoma resorption occurs even without surgical intervention [54]. This process is primarily mediated by activated microglia and recruited macrophages in the perihematomal region and involves erythrophagocytosis—the phagocytic clearance of red blood cells [55]. Strategies to enhance endogenous erythrophagocytosis is currently an active area of therapeutic investigation. Some molecular targets among the various signaling pathways implicated in this process have progressed to clinical trials.

Peroxisome proliferator-activated receptor gamma (PPARγ) has emerged as a key modulator of erythrophagocytosis and oxidative stress. Upon activation, PPARγ upregulates the expression of scavenger receptors (such as CD36) and antioxidant enzymes such as catalase, thereby promoting hematoma resolution and reducing oxidative injury [55]. PPARγ forms a heterodimer with the retinoid X receptor. Agonists of retinoid X receptor—such as bexarotene—have been shown to enhance erythrophagocytosis and accelerate hematoma clearance in experimental models [56]. However, despite these promising preclinical data, translation into clinical benefit remains challenging. The Safety of Pioglitazone for Hematoma Resolution in Intracerebral Hemorrhage trial, a phase II study of the PPARγ agonist pioglitazone, showed no significant improvement in mortality, cerebral edema, or functional outcomes at 90 days post-ICH [11].

Remote ischemic conditioning (RIC)—a maneuver involving repetitive inflation–deflation of a blood pressure cuff on a limb—has been shown to promote clot resolution in animal models through AMPK-dependent immune regulation [57]. In the RESIST RCT, which included 165 patients with ICH, RIC was not significantly associated with improved functional outcomes or 90-day mortality [57]. A larger phase III trial (NCT05609110) to further evaluate the efficacy of RIC in ICH is currently recruiting patients [58].

The breakdown of erythrocytes within the hematoma also releases hemoglobin and iron, which contribute to oxidative stress and trigger ferroptosis [59]. Therefore, iron chelation therapy represents another therapeutic strategy. The i-DEF trial—a randomized, double-blind, placebo-controlled study—found deferoxamine to be safe but ineffective in terms of improving favorable outcomes (mRS, 0-2) at 90 days. However, secondary analyses hinted at potential benefits at 180 days [13]. Further investigation of the benefits of iron chelation therapy is underway in the ongoing INTERACT-5 trial (NCT06763055).

In addition, extracellular hemoglobin released from lysed erythrocytes is directly neurotoxic. CD163, a scavenger receptor expressed by activated microglia and macrophages, mediates the endocytic uptake of hemoglobin–haptoglobin complexes [60,61]. Once internalized, hemoglobin is degraded by heme oxygenase (HO)-1 into iron, carbon monoxide, and biliverdin. This CD163/HO-1 pathway not only facilitates hemoglobin clearance but also exerts anti-inflammatory and antioxidant effects [60,62]. Recent human postmortem studies confirmed that this pathway is highly upregulated in the perihematomal region from day 8 to 15 post-ICH, which indicates the CD163/HO-1 pathway may represent a promising therapeutic target [63].

Additionally, cerebral lymphatic drainage may play a key role in eliminating red blood cells from the brain. Experimental ICH studies have demonstrated enhanced lymphatic function and meningeal lymphangiogenesis during hematoma resolution [64].

## 4 Stage 3: Mitigating Secondary Brain Injury

The secondary brain injury that occurs after ICH is driven by oxidative stress, iron toxicity, blood–brain barrier (BBB) disruption, cerebral edema, and neuroinflammation. These processes evolve over days to weeks, which presents a therapeutic window for neuroprotective interventions [65,66].

### 4.1 Pathophysiology of PHE

Early edema within the first few hours of ICH arises from osmotic shifts and extravasation of fluid due to vascular injury, serum protein leakage, and clot retraction. Delayed PHE typically develops over the next 3 to 4 days (Figure 2C) and is driven by vasogenic mechanisms due to BBB disruption and cytotoxic injury related to neuronal death, inflammation, and thrombin release.

### 4.2 Edema reduction strategies

Attempts to directly reduce cerebral edema have largely been unsuccessful. Corticosteroids, including dexamethasone, have shown no benefit on functional outcomes and were associated with increased mortality [36]. Mannitol, which is commonly administered for acute cerebral edema, lacks robust evidence of benefit. There are also concerns that mannitol can accumulate in injured brain tissue with BBB disruption, as in ICH [67]. This may result in a rebound effect if the brain mannitol concentrations exceed those in plasma [68]. Propensity score and multivariable analyses of the INTERACT2 cohort did not demonstrate improved functional outcomes for mannitol [69]. In fact, a meta-analysis suggested potential harm, as indicated by increased hematoma enlargement, higher mortality, and aggravated edema when mannitol was administered within 24 hours of ICH onset [70]. Current guidelines recommend reserving mannitol for patients with clinical or radiological signs of significant cerebral edema and raised intracranial pressure, typically as a bridge to surgery [35]. The MACE-ICH trial (ISRCTN15383301), which is currently enrolling patients within 72 hours of onset, aims to clarify the benefit of mannitol in acute ICH [14].

Glibenclamide, which inhibits the sulfonylurea receptor and translocation of aquaporin-4 to the plasma membrane, has shown promise to reduce edema in ischemic stroke [71]. In ICH, the GATE-ICH trial reported a trend toward improved 3-month functional outcomes and significant reductions in PHE parameters in the glibenclamide group; however, treatment was associated with a higher incidence of asymptomatic hypoglycemia [15].

### 4.3 Targeting inflammation and immune responses

PHE is closely linked to perihematomal inflammation, which has become a major therapeutic target. Microglial activation begins with an early M1-predominant proinflammatory response that promotes BBB disruption, extracellular matrix degradation, and recruitment of reactive astrocytes and T helper cells. This response gradually transitions over days to a reparative M2-predominant phenotype that facilitates hematoma clearance, edema resolution, and tissue repair [72]. Modulating this shift by dampening early M1 activity and promoting timely M2 responses offers a potential therapeutic avenue. However, post-ICH neuroinflammation is a complex, continuous process—rather than a simple M1/M2 dichotomy; thus, the effects of targeted interventions could be beneficial or detrimental depending on the timing and the biological processes involved [54]. Careful temporal precision is therefore essential when designing experimental and clinical strategies.

Several anti-inflammatory agents have been investigated in ICH. Minocycline is a central nervous system–penetrant tetracycline that exerts inhibitory activity on proinflammatory microglia/macrophages and iron-chelating properties and is an inhibitor of matrix metalloproteinases. Early-phase trials (Minocycline in Acute Cerebral Hemorrhage and Minocycline and matrix metalloproteinase inhibition in acute intracerebral hemorrhage) showed no improvement in functional or radiological outcomes, despite reducing serum matrix metalloproteinase 9 levels [16,17]. Ongoing studies (eg, NCT05630534) aim to reassess the efficacy of minocycline. Retrospective data suggest minocycline is generally well-tolerated and may reduce the recurrence of ICH in patients with clinically aggressive cerebral amyloid angiopathy [73]. A dedicated phase I/II trial (BATMAN, NCT05680389) is currently underway [18].

Anakinra, an interleukin (IL)-1 receptor antagonist, was evaluated in the small, early-phase BLOC-ICH trial, which demonstrated reduced systemic IL-6 levels but no significant effect on PHE or functional outcomes. However, this study was primarily a feasibility and safety trial with only 25 participants and was thus underpowered to assess clinical efficacy [19]. The phase II ACTION trial, targeting IL-1β within 8 hours, is ongoing [74]. Colchicine exhibits broad anti-inflammatory effects. The phase II CoVasc-ICH trial (NCT05159219) has been completed, and the preliminary results are awaited; the phase III CoVasc-ICH 2 trial (NCT06587737) is scheduled to begin in 2025. Fingolimod, a sphingosine-1-phosphate receptor modulator, limits lymphocyte trafficking into the central nervous system. Small RCTs demonstrated reduced edema and improved early neurologic outcomes in patients with small-to-moderate deep ICH [75]. A phase I trial (FITCH; NCT04088630) has been completed, with results pending. Siponimod, another S1PR modulator, was evaluated in a phase II trial (NCT03338998), which was terminated early during the COVID-19 pandemic; no significant differences in edema were found in the limited sample.

Celecoxib, a selective COX-2 inhibitor of nonsteroidal anti-inflammatory drugs, has also been investigated as a potential therapy in ICH. The ACE-ICH pilot trial reported that celecoxib (400 mg twice daily for 14 days) significantly reduced PHE compared with control therapies [20]. Supporting this, a recent retrospective study found that nonsteroidal anti-inflammatory drug use within 4 weeks after ICH was associated with lower 1-year mortality compared with no use [76]. To build on these findings, a phase II prospective trial (NCT05434065) is currently evaluating the effects of earlier celecoxib administration, within 6 hours of symptom onset. Finally, statins are known to have anti-inflammatory properties. Although observational studies suggest mixed effects on edema and functional outcomes, registry data (*n* = 1275) indicated that initiating statins post-ICH was associated with increased peak edema, whereas continuation of prior statin therapy had no significant effect [[77], [78], [79]]. Some of the trials are ongoing, including statin use in intracerebral hemorrhage (phase III; NCT03936361) and statin for neuroprotection in spontaneous intracerebral hemorrhage (phase II/III; NCT04857632).

### 4.4 Antioxidants and free radical scavengers

Oxidative stress is a major contributor to secondary injury [54]. Edaravone, a potent free radical scavenger, was evaluated in meta-analyses, which showed no mortality benefit when administered within 7 days and continued for 14 to 30 days [80]. A dose-ranging phase I/II trial is ongoing (NCT05953103). Another reactive oxygen species scavenger, *N*-acetylcysteine with selenium, was evaluated in a randomized trial. The findings suggested significantly reduced PHE volume, but the generalizability of this study was limited due to the heterogeneous population, which included secondary ICH [81].

### 4.5 Other experimental neuroprotective strategies

A variety of other agents such as microRNAs [82], exosomes [83], nicotine [84], melatonin [85], and N-methyl-D-aspartate receptor antagonists [86,87], as well as nanoparticle-based treatments [88], have demonstrated neuroprotective effects in preclinical studies, but robust clinical evidence is lacking [54]. Stem cell transplantation has shown encouraging results in small-scale studies [[89], [90], [91]], but larger, well-designed clinical trials are needed to confirm safety and efficacy. Combination therapies targeting multiple pathways—such as hematoma clearance, inflammation, and oxidative stress—and novel interventions such as enhancing glymphatic drainage remain investigational.

## 5 Clinical Implications: From Pathophysiology to Precision Medicine

The persistent gap between radiological success and functional recovery in ICH trials highlights the radiological–clinical mismatch. This issue is frequently attributed to a threshold effect, where statistically significant reductions in HE or PHE fail to reach the magnitude required to preserve long-term neural connectivity. Such translational failures are further driven by patient heterogeneity and temporal misalignment. While stage 1 interventions must be administered during an ultraearly window, stage 3 modulation faces a more complex temporal paradox: agents must be precisely timed to suppress early neurotoxicity without impairing the delayed, endogenous phagocytic processes essential for hematoma resolution. Identifying the optimal window for these interdependent processes remains a critical gap in current trial designs.

Moreover, the 3 therapeutic stages should be viewed as a biologically interdependent continuum. A failure in ultraearly hematoma stabilization (stage 1) directly exacerbates the iron-mediated toxicity and inflammatory cascades that drive late-stage secondary injury (stage 3). Consequently, the future of ICH management lies in a time-sensitive bundle-of-care approach. This model emphasizes the simultaneous application of hyperacute hemostasis, early surgical intervention where appropriate, and sequential, precisely timed immunomodulation to halt the pathological cascade. Furthermore, the clinical utility of this bundle depends on the success of tailoring these interventions to specific patient phenotypes. Precision frameworks suggest that while lobar ICH (30-80 mL) derives the most clear functional benefit from MIS (as seen in the ENRICH trial), deep large hypertensive bleeds may require survival-oriented strategies such as decompressive craniectomy, whereas anticoagulant-associated cases necessitate immediate specific pharmacologic reversal.

## 6 Conclusion

The management of ICH is gradually evolving from therapeutic nihilism toward optimism. While no single intervention has achieved dramatic functional benefits, incremental advances in ultraearly care, MIS, and modulation of inflammation are reshaping the therapeutic landscape. By incorporating multimodal and phenotype-specific interventions, clinicians are now moving forward to proactively improve the trajectory of patient recovery in ICH.
